# Making a new bromo-containing cellulosic dye with antibacterial properties for use on various fabrics using computational research

**DOI:** 10.1038/s41598-023-36688-y

**Published:** 2023-06-21

**Authors:** Fatma N. El-Shall, Asmaa M. Fahim, Sawsan Dacrory

**Affiliations:** 1 Dyeing, Printing and Textile Auxiliaries Department, National Research Centre, P.O. Box 12622, Dokki, Cairo, Egypt; 2 Green Chemistry Department, National Research Center, P.O. Box 12622, Dokki, Cairo, Egypt; 3 Cellulose and Paper Department, National Research Centre, Giza, 12622 Egypt

**Keywords:** Chemistry, Materials science

## Abstract

The reaction of cyanoethyl cellulose with para-bromo diazonium chloride resulted in the creation of a novel bromo-containing cellulosic (MCPT). The dispersion stability of MCPT has been improved by its dispersion into 1% waterborne polyurethane acrylate (WPUA). TEM, particle size, and zeta potential were used to track the dispersion stability of aqueous MCPT and MCPT in 1% WPUA and particle size. The prepared MCPT has been utilized as a unique green colorant (dye) for the printing of cotton, polyester, and cotton/polyester blend fabrics using a silkscreen printing technique through a single printing step and one color system. Color improvement has been achieved by printing different fabrics with a printing paste of MCPT dispersed in 1% WPUA. The MCPT and MCPT in 1% WPUA printed fabrics were evaluated for rubbing, light, washing, and perspiration fastness, UV blocking activity, and antibacterial activity. These findings were established through structural optimization at the DFT/B3LYP/6-31 (G) level and simulations involving several proteins.

## Introduction

The textile sector ranks as the second-largest polluter in the world, and synthetic dyes contribute significantly to this environmental pollution. These dyestuffs have extremely dangerous effects on both the environment and human health, including carcinogenicity and mutagenicity. As a result of increased global awareness of environmental issues and a growing focus on cleaner and greener products and technologies, the demand for sustainable dyestuffs for textile coloring has been progressively expanding over the last few decades^1–4^. One of these solutions is the use of natural dyes as an alternative to synthetic dyes. Natural dyes were utilized in the apparel, cosmetic, pharmaceutical, and food-related sectors. Despite all of the advantageous features of natural dyes, such as their non-polluting effect, 100% biodegradability, wide range of colors and shades, and skin safety, they have a number of drawbacks, such as high cost, poor brightness, limited availability due to changing planting seasons, and solubility issues^5,6^.

Ultimately, the fashion and textile industries are actively searching for promising coloring materials and techniques. Therefore, more effort is required to develop new and innovative approaches that can fill these gaps, and some novel sustainable dye alternatives with low or even zero environmental impact should be developed^7–9^.

Here are a few of the more inventive approaches applied to the textile coloring sector:

Powder dyes from textile fibers: The pigments are generated by recovering used clothing fibers into a fine powder that may be used as a textile dye. It may be used to generate long-lasting color shades with a washed-out appearance^10^. Natural or engineered microorganisms: A either synthetic or natural biological system was utilized to color the garment by fixing the dye-producing microorganisms directly onto the textile material, that may minimize the consumption of water by as much as ten times^11^. Innovative dye and auxiliaries and digital printing. Hybrid pigment: developed from dye that has been chemically bonded to a special polymer particle that interacts with textile fibers^12,13^.

Furthermore, Waste-derived natural polymers attract considerable attention to achieve zero waste because they are renewable, abundant, biodegradable, and nontoxic^14–16^. In particular, cellulose, which can be obtained from renewable agriculture waste, is one of the most appealing candidates owing to its biodegradability, biocompatibility, and low cost. Moreover, its unique structure provides cellulose with the ability to form intramolecular hydrogen bonding, insolubility in conventional solvents, and susceptibility to undergo modification with functional groups, which render it suitable for a wide range of applications^17–20^. The utilization of Nano-fibrillated cellulose (NFC) hydrogel and oxidized cellulose nanocrystals (ONC) as superior color carriers for textile dyes has been demonstrated in the dyeing of cotton, denim, and wool fabric by reactive dyes. This approach has the potential to minimize dyeing waste water by up to 60%, in addition to enhancing color depth and fastness properties^8,21–23^. Moreover, attempts have been made to generate colorants based on cellulose derivatives (azo cellulose derivatives) from cyanoethyl cellulose derivatives that exhibit particularly attractive physical, chemical, and multifunctional properties (UV blocking activity, antimicrobial, and anticancer) depending on the degree of substitution^24–29^.

On the other hand, blended fabrics combine the distinct properties of cotton fabrics of natural origin (wear comfort, reduced pilling, and water absorption) and polyester fabrics of synthetic origin (abrasion resistance, tensile strength, and dimensional stability)^30,31^. However, to color this type of textile fabric, two different classes of dyes must be used under different coloration conditions, which complicates the coloring procedure. To solve this problem, different treatments, finishing methods, new structures, and fabric surface modifications are being explored. Furthermore, to achieve sustainability, reducing the environmental pollution load as well as the cost, energy, and chemicals used in textile processes is an essential but challenging task^32–34^.

In this study, we synthesized and characterized bromopyrazolo[5,1-c][1,2,4]triazin-3-yl as a novel Bromo-containing azo cellulosic dye (MCPT) by coupling cyanoethyl cellulose with diazonium chloride to yield a hydrazone, which was then cyclized into pyridine. Furthermore, the effect of synthesized waterborne polyurethane acrylate (WPUA) on the dispersion stability and particle size of MCPT was investigated. The utilizing of MCPT to impart color to cotton, polyester, and cotton/polyester (Co/PET) blend fabrics via silkscreen printing technique, as well as the evaluation their fastness properties, antimicrobial behavior, and ultraviolet (UV) shielding activity, are discussed. A docking study using different proteins and theoretical calculations revealed the formation of hydrogen bond interactions between the dye and the fabric surface, which confirmed the experimental results.

## Methods

### Instruments

FT-IR was analyzed via Shimadzu FT-IR 8101 PC spectrum and the 1HNMR, 13CNMR were analyzed in DMSO solvent at 300 MHz on a Varian Mercury using TMS as an internal standard. UV–Vis spectrophotometer measurements were carried out by the JASCO V-730 UV–visible/NIR double-beam spectrophotometer, Tokyo, Japan. The scan was performed from 200 to 800 nm in DMSO as solvent. The SEM photos were detected using JEOL JXA-840A electron probe microanalyzer, and the samples were air-dried before captures were at voltage of 10–15 kV using FEI IN SPECTS Company, Philips, Holland. Moreover, TEM analysis were taken with a high-resolution JEOL JEM-2100/Japan. The samples were deposited from an aqueous dilute dispersion on a micro grid covered with a thin carbon film (≈ 200 nm). The Particle Sizing Systems (Santa Barbara Inc., California, USA) were used to estimate the particle size and zeta potential (ζ) of samples. The color strength (K/S) of printed fabrics was determined using the Mini ScanTM XE Hunter-Lab Universal Software, which is based on the equation of Kubelka–Munk: K/S = (1 − R)2/2R, where K denotes the absorption coefficient, S denotes the scattering coefficient, and R denotes the fraction of light reflected at a wavelength of minimum reflectance or maximum absorbance. CIE lab color parameter L* specifies the sample's brightness, a* and b* are chromaticity coordinates specifies the sample's redness-green and yellowing-bluish shift respectively^35^. The fastness property of washing, rubbing, perspiration, and light is evaluated using standard methods^36^. The UV protection factor (UPF) was calculated using a UV-Shimadzu 3101-PC-Spectrophotometer and the Australian/New Zealand Standard (AS/NZS-4399-1996), a UPF value that less than 20 indicates poor protection, 20–29 indicates good protection, 30–40 indicates very good protection, and > 40 indicates excellent protection.

### Reagents

Microcrystalline cellulose, CH=CH–CN, NaN_3_, EtOH, CH_3_COOH, DMSO, Polyethylene glycol supplied were obtained from Fluka company. Hydroxyethyl acrylate (HEA) supplied by Degussa, Germany. Dibutyltin dilaurate, (DBTDL), isophorone diisocyanate and sorbitol were acquired via across Chemical Co, used as received. Cotton fabric (100% Scoured, bleached, plain weave, 140 g/m^2^), polyester fabric (100% white) plain weave (149 g/m^2^) and cotton/polyester blend (CO/PET) fabric (50/50%, 135 g/m^2^) were providing through Misr Company for Spinning and Weaving, Mehalla El-Kubra, Egypt. Bercolin CPK, thickener supplied by Berssa-Turkey.

### Reactivity of cellulose (1) with CH=CH–CN (2)

Microcrystalline cellulose (MCC) (1) (3 g, 0.018 mol) was mixed in 60 ml of sodium hydrozide solution (10%) at − 10 °C for 24 h to give a clear solution then the add the CH=CH–CN (**2**) (9 ml, 0.137 mol) dropwise to the solution of microcrystalline cellulose with stirring at 5–10 °C for 6 h. Lastly, the solution was neutralized with CH_3_COOH, wash away and crystallized with ethanol, and then dried to afford (MCEC) (**3**)(white solid) in 85% yield and the analyzed: FT-IR(KBr) ν max/cm^−1^: υ = 3461(OH), 2940(CH), 2265(C≡N), 1HNMR (DMSO-d6): 2.75(t, 2H, H2C), 3.07–3.83(m, 14H, HC-glucose moiety), 4.37–4.66(t, 2H, H2C), 5.47–5.65(t, 1H, HC), ^13^C NMR (DMSO-d6):δ 18.69(CH2), 24.3(CH2), 66.32(CH2), 84.11(CH), 91.6 (CH), 103.5 (CH), 120.1(C≡N) Furthermore, DS degree of substitution of cyanoethyl content values of the MCEC was calculated through N_2_ content with this Eq. (1)^33^

1 $$DS = \frac{(162*N\% )}{{(1.400 - 53*N)}}$$

### Reactivity of MCEC (3) with diazonium chloride of 3-bromo-5-methyl-1H-pyrazole

Solution of MCEC (3) (2.55 g, 10 mmol) with diazonium Chloride of 3-bromo-5-methyl-1H-pyrazole (10 mmol) dissolved in 30 ml pyridine, and addition was dropwise at 0–5 °C for half hour, then stirred for 4 h, and keep in fridge for 12 h, and diluted with water and formed solid collected and filtered off washed with water then crystallized with mixture EtOH/DMF afforded the corresponding hydrazone derivatives **4a**.

### Cyclization of the hydrazones derivative

Solution of hydrazone derivative **4a** (1 mmol) in pyridine (15 ml) was heated for 6 h, cooled and filtered off, washed with EtOH, and crystallized with DMF/H_2_O to give MCPT(5a) ***(2R,3R,4S,5R,6S)-6-(((4-amino-7-bromopyrazolo[5,1-c][1,2,4]triazin-3-yl)methoxy)methyl)-2,5-dimethoxytetrahydro-2H-pyran-3,4-diol(5):*** pale brown, yield = 88%;m.p = 220–222 °C, FT-IR (KBr): ν 3320(OH), 3211(NH_2_), 2990 (CH_2_), 1510(C=C), 1246(C–O–C) cm^−1^. UV-Absorption band:λ = 370.5 nm, ^1^H-NMR (DMSO): δ 3.54(m, CH_2_, glucose), 4.052(m, CH_2_), 5.118 (m, H, CH_2_), 6.113 (H,s, NH_2_, D_2_O exchangeable), 7.5 (m, H, CH), 9.27 (1H, s, CH=), ^13^CNMR(DMSO), δ 55.2(CH_2_), 82.2(CH_2_), 111.5 (CH),116(CH), 118(CH),129(CH),149(CH), 157(CH),

### Synthesis of polymer waterborne polyurethane acrylate (WPUA)

The synthesized of Waterborne polyurethane acrylate (WPUA) was obtained through a poly-addition reaction of polyethylene glycol (6000 g/mol), isophorone diisocyanate, sorbitol as saturated hydrophilic chain extender (improve the adhesion between the PUA film and substrate) and the hydroxyethyl acrylate as UV reactive capping agent under inert atmosphere as the following^37^: In a flask with three necks fitted with a stirrer, a thermometer, and a reflux condenser under nitrogen, a calculated quantity of polyethylene glycol (6000 g/mol) and sorbitol (11:1) was added as in 70% acetone and left for approximately 1 h to ensure the full mixing of the reaction elements.

Over an hour, an appropriate quantity of IPDI with 0.05 (w/w) DBTDL as a catalyst was carefully added into a reactor running at 40 °C. To ensure a satisfactory reaction rate while avoiding gelation, the reaction solution was stirred regularly for an extra hour at 40 °C.

To cap the entire terminal NCO group, an appropriate quantity of HEA was progressively introduced into the reaction solution over 1 h at 60 °C, and then the reaction medium was stirred constantly for another 2 h frequently at 60 °C. The end result was a clear solution of polyurethane acrylate.

### Printing paste recipe, technique, and fixation

The paste used in fabric printing was made with 4% synthesized MCPT or MCPT in 1% synthesized WPUA and 1% ammonium persulfate and a synthetic thickener of 4 g/100 ml. The prepared printing pastes were homogenized and used for silk screen printing of the fabrics. The printed fabrics were thermo-fixed in an automatic thermostatic oven for 5 min at 160 °C before being washed in cold water, hot water, and then cold water again to remove any excess thickener or unreacted materials.

### Antimicrobial activity

Antibacterial activity of printed fabrics of (MCPT) and (MCPT dispersed in 1% of WPUA) against *Pseudomonas aeruginosa* and *Salmonella Typhimurium* (−Ve bacteria), *Bacillus subtilis*, and *Enterococcus facials* (+Ve bacteria) was studied in vitro using nutrient agar medium. The tested sheets were located on the surface of the solidified media plates, and the plates were raised at 37 °C for 16–24 h. The activities were calculated by comparing the inhibition zone (IZ) compared to the test antibacterial strain to standard.

### Molecular docking studies

Docking of cellulosic derivatives and different fabrics were analyzed through MOE program^38^ and determine their energy affinity, bond length and attached amino acids, with different geometry which support with RMS gradient of 0.01 Å, and examined with protein’s called Crystal structure of Escherichia coli MenB in complex with substrate analog, OSB-NCoA (**PDBID:**3t88)^39^, and Crystal structure of the tyrosine phosphatase Cps4B from Streptococcus pneumoniae TIGR4 (**PDBID:**2wje)^40^. Ten docking simulation were track via standard parameters and the confirmations were selected and built on the procedure of total statistics, E configuration, and appropriate with the related amino acids in pocket for each protein.

### DFT studies

The DFT/B3LYP/6-31(G) level were used and optimized utilized the Gaussian 09 program^41^ and its majority benefit DFT approaches that provide increase the computational accuracy without increasing computation time. All chemicals were imagined via Gauss-View interface^42^. The basic parameters were estimated with Physical parameters retrieved from files^43^.

## Results and discussion

### Preparation and analysis

**Figure 1:**
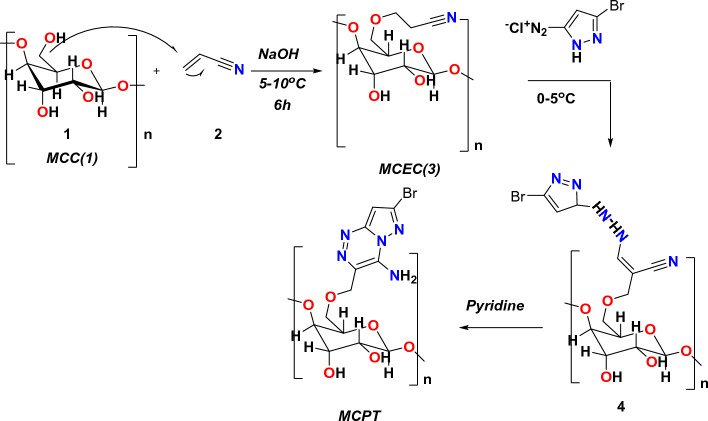
Synthesis procedure of **MCPT** cellulosic dye.

Microcrystalline cellulose (**1**) react with CH=CH–CN in the presence of sodium hydroxide solution at little temperature furnished the corresponding microcrystalline cyanoethyl cellulose (MCEC) through Michael reaction and the number of nitrogen substitution with DS = 1.5 as revealed in Fig. 1. FT-IR of dye **MCEC (3)** displayed OH absorption band at 3461 cm^−1^ and C≡N band at 2265 cm^−1^, ^1^H NMR presented signals at δ 2.75 and 4.37 owing to CH_2_ group, besides glygose protons in the region 3.07–3.83 ppm; correspondingly. The behavior of acetamide **3** with a diazonium salt to give Br-hydrazones **4**. The IR spectra of the isolated hydrazones exhibited bands at 3520–3121 cm^−1^ corresponding to NH function groups and a C≡N absorption band in the region 2500 cm^−1^, besides the strong C=O bands in the region 1720–1688 cm^−1^. Br-Hydrazone **4** underwent an intramolecular cyclization in pyridine via Michael-type addition of the endocyclic NH of the hydrazones 4a to the triple bond of a C≡N function group to give the MCPT(5a) as displayed in Fig. 1. MCPT revealed FT-IR spectrum absence of C≡N group and the presence of NH_2_ at 3360 cm^−1^ and C=O groups extending from 1671 to 1611 cm^−1^, however, its ^1^H NMR exhibited the attendance of D_2_O exchangeable proton in the area from 6.5 ppm as a result of the NH_2_ at 7.35 ppm due to pyrazolo[5,1-c][1,2,4]triazin-3-yl protons^19^.

### Fourier-transform infrared and UV spectroscopy

**Figure 2:**
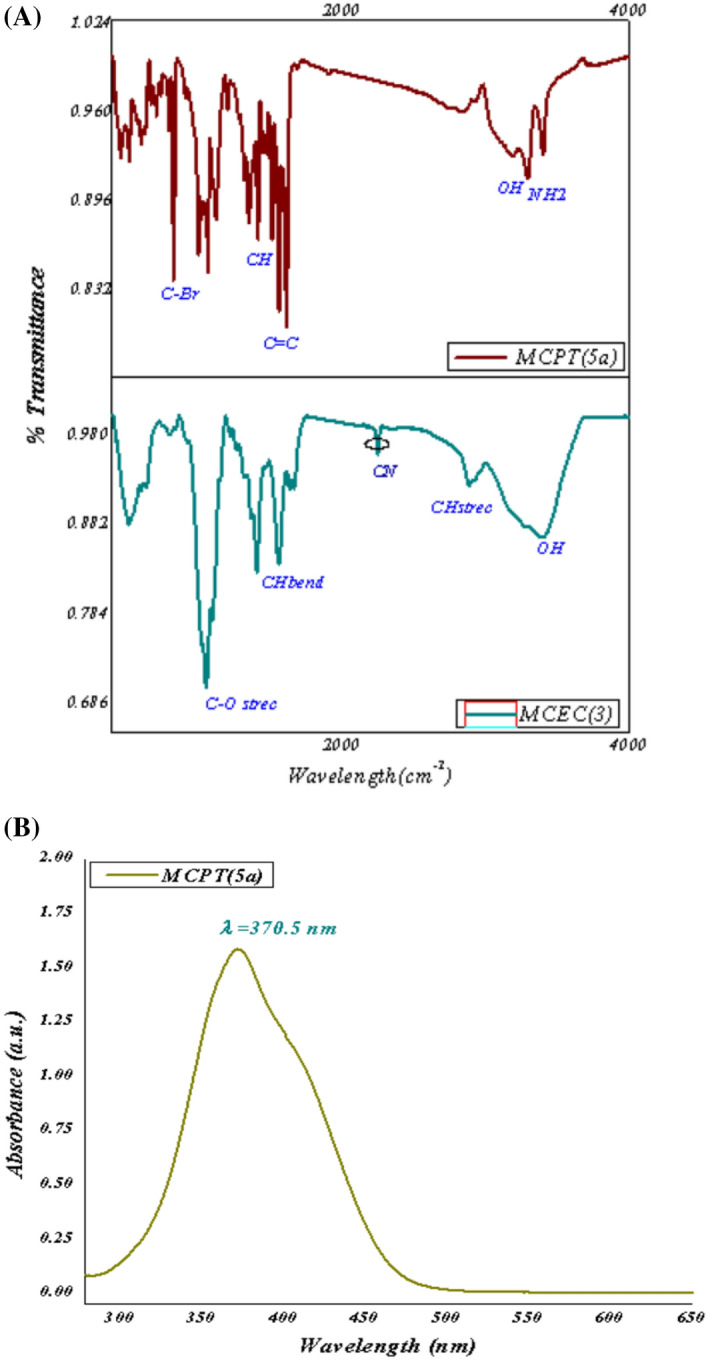
(**A**) FT-IR of the MCEC and Br-cellulosic dye (MCPT). (**B**) UV-absorption of the (MCPT).

FT-IR groups of produced heterocyclic cellulosic (**MCEC**) and **(MCPT)** were displayed in Fig. 2A. It observed that FT-IR frequencies of manufactured (**MCEC**) were demonstrated in Fig. 2A and exhibited the attendance of absorption band for hydroxyl, CH groups at 3461 cm^−1^ and 2940 cm^−1^ for **MCEC**. Additionally, in the stretching absorption band of CH υ = 2940 cm^−1^, the C≡N at **MCEC** seems at υ = 2265 cm^−1^ and also CH aliphatic at υ = 2931 cm^−1^ attributable to the chemical reaction of **MCC** with C≡N in basic condition (**MCEC**) display different characteristic absorption bands at 2265 cm^−1^ and 2928, 1574 and 1411 cm^−1^ as a result of stretching and bending CH aliphatic band, individually (Fig. 2A). Besides, the FT-IR classification of **MCPT** presented the hydroxy group of glycose ring at 3320–3345 cm^−1^; individually, although their NH_2_ group takes abroad rang with OH of glycoside rings and showed at placing 3211–3205 cm^−1^, similarly the double bond of the fused cyclic ring presented absorption band at 1510–1499 cm^−1^and C–O–C presented the band at 1246–1233 cm^−1^; individually.

Moreover, the UV spectra of MCPT showed maximum absorptions band at λ = 370 nm as displayed in Fig. 2B and this indicated the conjugation of MCPT with alternating double and single bonds in (2R,3R,4S,5R,6S)-6-(((4-amino-7-bromopyrazolo[5,1-c][1,2,4]triazin-3-yl)methoxy)methyl)-2,5-dimethoxytetrahydro-2H-pyran-3,4-diol which characteristically absorb light in the visible region.

### SEM analysis

**Figure 3:**
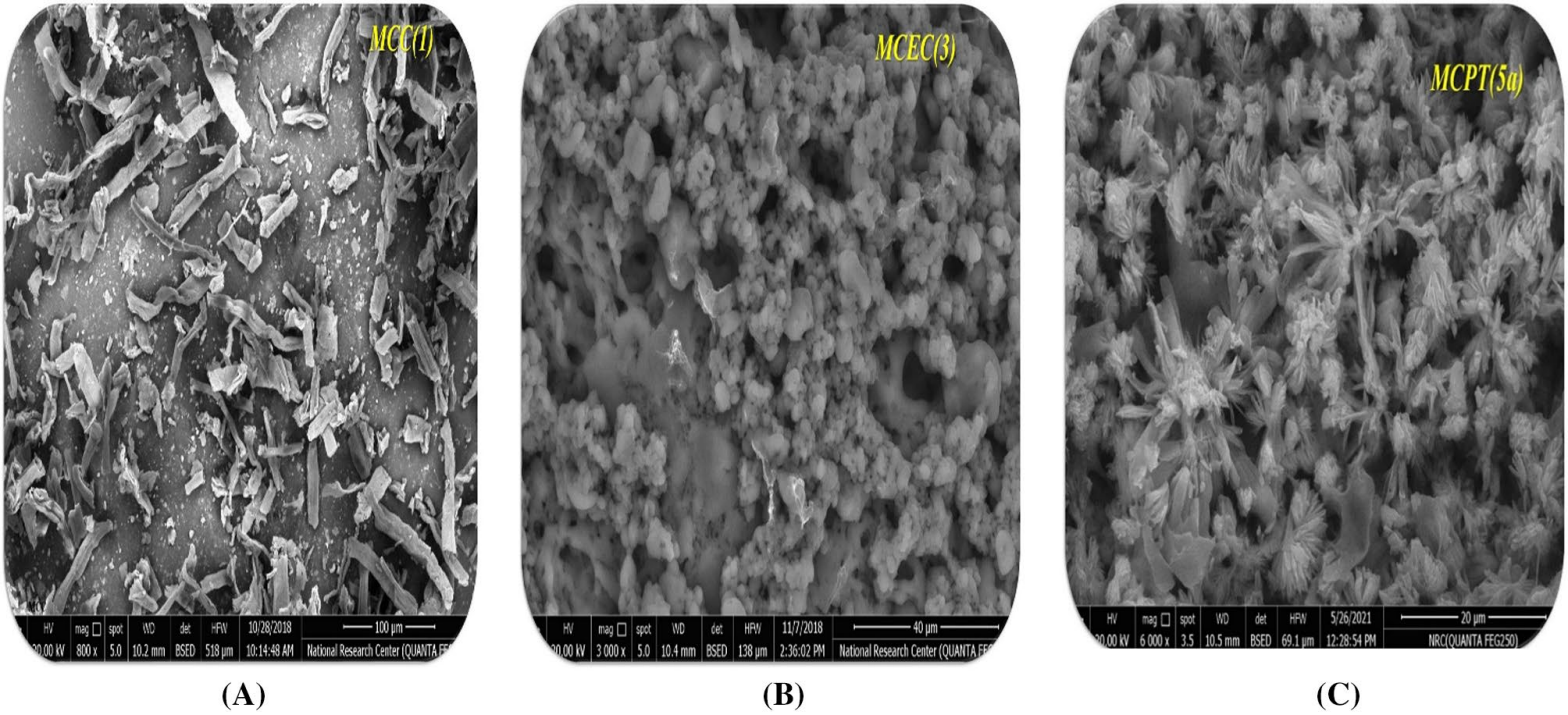
SEM of the MCEC, MCEN, and MCPT.

SEM examination of surface morphology of **MCC,** which displayed the scratched surface of cellulose, although the behavior of **MCC** with C≡N which gave the **MCEC** with surface sponge morphology foams because of occurrence cyanide group and make growth on the surface of cellulose and extra with hydroxyl group of glycoside ring as demonstrated in Fig. 3A,B,C. Additionally, MCEC reactivity with pyrazole gave the corresponding Br-4-aminopyrazolo[5,1-c] [1, 2, 4] triazincellulose derivatives (MCPT)(**5a**). MCPT presented the surface morphology of surface was fishes scratched and Br presence attached to benzene ring manufacture withdrawing character and modification all cellulose surface.

## Interaction of WPUA and MCPT

### synthesis of waterborne polyurethane acrylate (WPUA) and dispersion of the MCPT on WPUA

**Figure 4:**
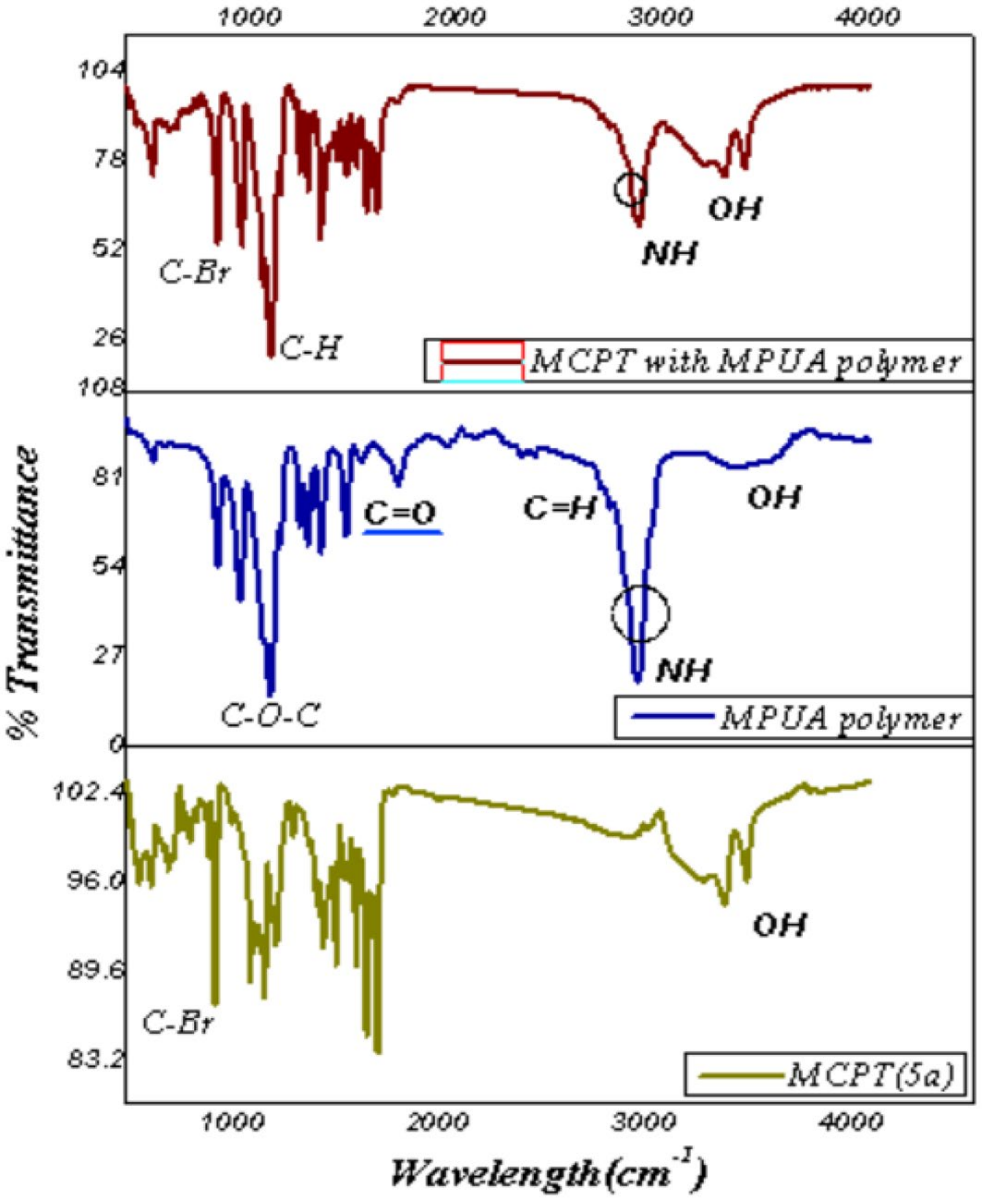
FT-IR of the synthesized MCPT, WPUA, and MCPT with1%WPUA.

The WPUA was synthesized by poly-addition of polyethylene glycol (6000 g/mol) with isophorone diisocyanate, the polymer chain was extended using sorbitol as hydrophilic chain extander to improve the adhesion forces of the polyurethane films, and the reaction was further reacted with hydroxyethyl acrylate, which acts as a UV reactive capping reagent in the presence of (DBTD) (Di-butyl tin dilaurate) as a catalyst under nitrogen atmosphere. The spectral characterization of WPUA was confirmed through FT-IR investigation, as displayed in Fig. 4 as OH_strecting_ and N–H broadband at 3361 cm^−1^, st CH_2_, CH_3_, CH at 2900 cm^−1^, C=H _Stretching_ at 2737 cm^−1^, C=O s_tretching_ at 1171 cm^−1^, st C-N_Stretching_ at 1338 cm^−1^, C–O–C _Stretching_ at 1094 cm^−1^ and δ: N–H & δ: CH_2_ at 1540 and 1496 cm^−1^; respectively as displayed in Fig. 4.

**Figure 5:**
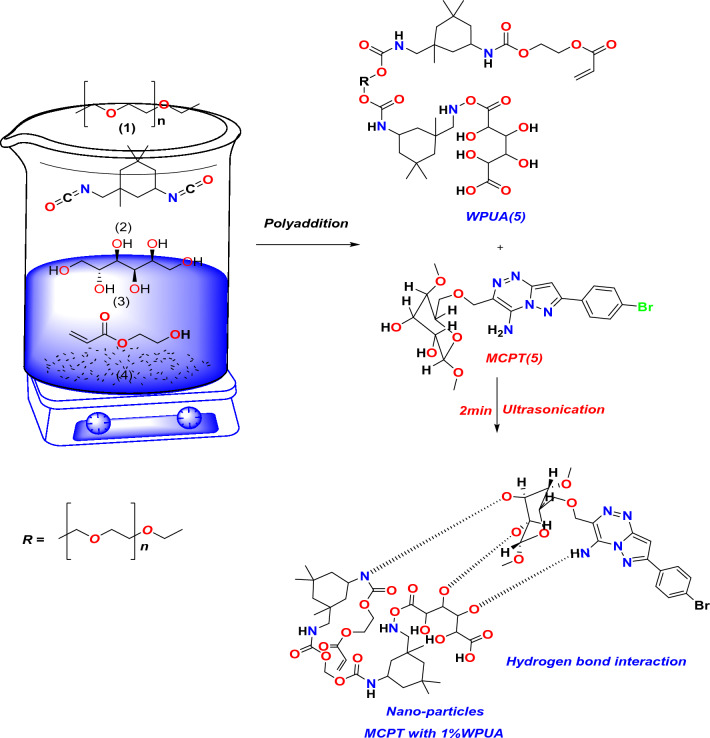
Plausible interaction between 1% of WPUA with MCPT using ultra-sonication.

Furthermore, the dispersion of Br cellulose MCPT in 1% WPUA was utilized by the ultra-sonication probe for 2 min as a green tool to make a complete dispersion and separate connected Nano particles^44^. The plausible interaction between MCPT and WPUA occurred through hydrogen bond interaction, as displayed in the proposed mechanism Fig. 5. The FT-IR of MCPT in (1%) WPUA showed the different absorption bands at OH stretching vibration at 3340 cm^−1^ and also the NH group showed in same range at 3330 cm^−1^, C=H the aromatic of phenyl ring appears at 2980 cm^−1^, CH bending showed at 1460 cm^−1^, C–Br showed at 810 cm^−1^ which showed the intramolecular hydrogen bond interaction between OH of MPUA polymer with MCPT and amino group which reduced at showed in Fig. 4.

Additionally, as WPUA particles are well dispersed in aqueous media as dual colloidal systems, waterborne polyurethanes (WPUA) appear to be becoming more common as eco-friendly alternatives to conventional polyurethanes as dispersing and/or binding agents^45,46^. The dispersion stability and particle size of MCPT were tracked among MCPT aqueous dispersion and aqueous 1% WPUA.

### TEM and DLS investigation

**Figure 6:**
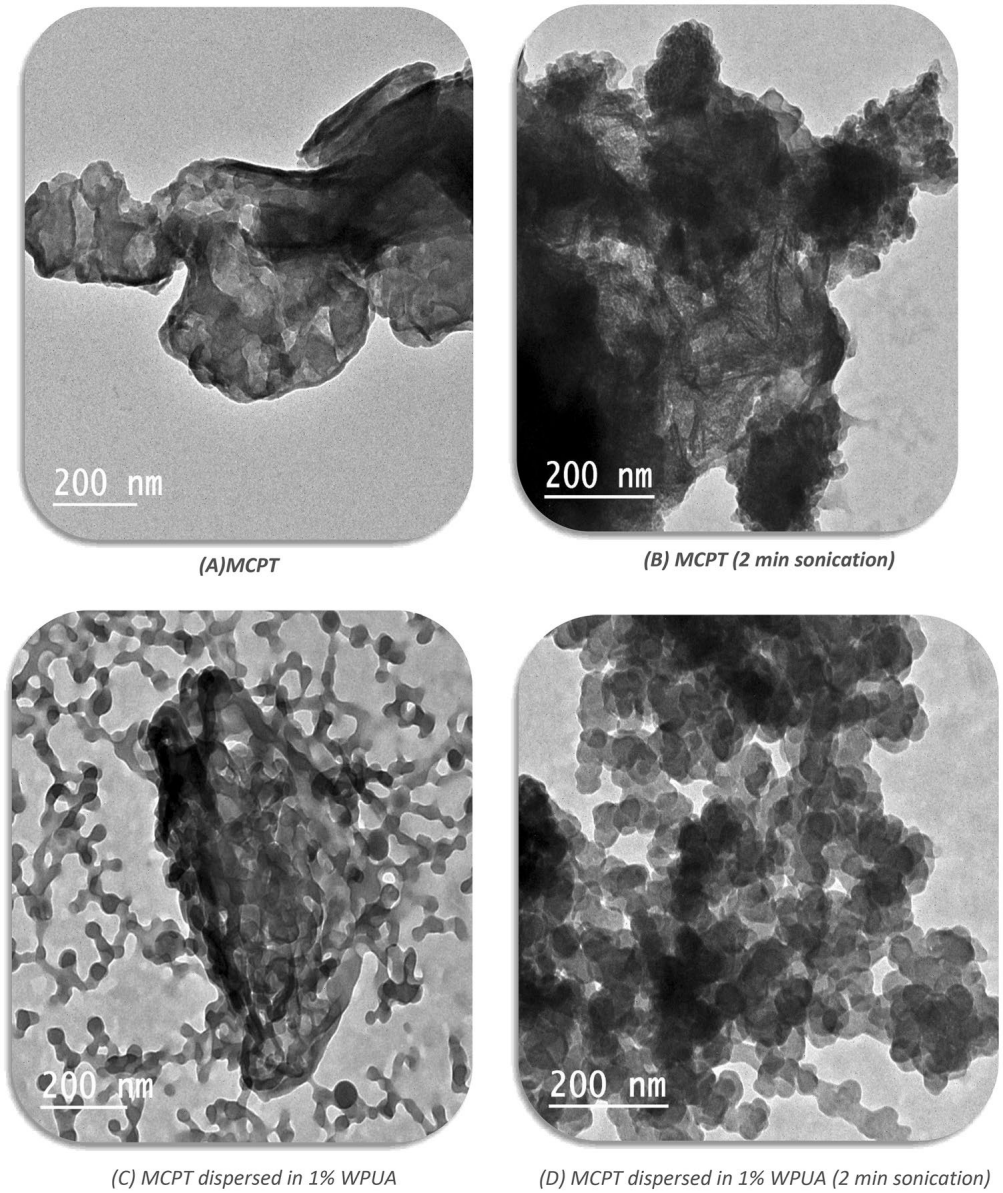
(**A**)**–**(**D**) TEM images of MCPT dispersions in polymer.

Figure 6A,B TEM images of MCPT aqueous dispersion demonstrated that the particles clumped together and accumulated even when subjected to ultra-sonication due to the particles' large surface area as a result of hydrophobic interaction^47^. Furthermore, MCPT dispersion in 1% WPUA interacted with the polymeric layer of WPUA via hydrogen bond formation. This interaction's steric repellent effect overcame the particles' tendency to collect, flocculate, or re-agglomerate, resulting in improved dispersion stability^47,48^. As a result, Fig. 6C showed Nano-sized well dispersed MCPT particles. Smaller particles with uniform and homogeneous distribution with a core shell-like shape appeared in the Fig. 6D as a result of the effect of ultrasonic waves on the MCPT in 1% WPUA dispersion.

Furthermore, the TEM results can be supported by the results obtained from the DLS parameters as shown in Table 1 [mean diameter (*Ø*), variance (*PI*), standard deviation (*Sd*), and average zeta potential (*ζ*)]. The zeta potential value designated the physical stability of the suspensions. It is known that the higher the negative or positive value of the zeta potential, the greater the stability of the suspension^49^. The outcomes displayed that the highest values of the zeta potential were recorded with the dispersion of MCPT in 1% WPUA and MCPT in 1% WPUA (2 min sonication), which indicates the sufficient stability of these dispersions.

**Table 1:** DLS parameters [mean diameter (Ø), variance (PI), standard deviation (Sd) and avg. zeta potential (**ζ**)].

Parameter	MCPT	MCPT with (2 min sonication)	MCPT in 1% WPUA	MCPT in 1% WPUA (2 min sonication)
Ø [nm]	1636.7	222.3	170.9	152.0
PI	0.375	0.064	0.453	0.412
Sd [nm]	1001.7 nm (61.2%)	56.2 nm (25.3%)	115.0 nm (67.3%)	97.6 nm (64.2%)
ζ [mV]	− 1.32	− 3.72	− 23.54	− 34.97

## Printing application

### Coloring properties of printed fabrics with MCPT and WPUA

**Figure 7:**
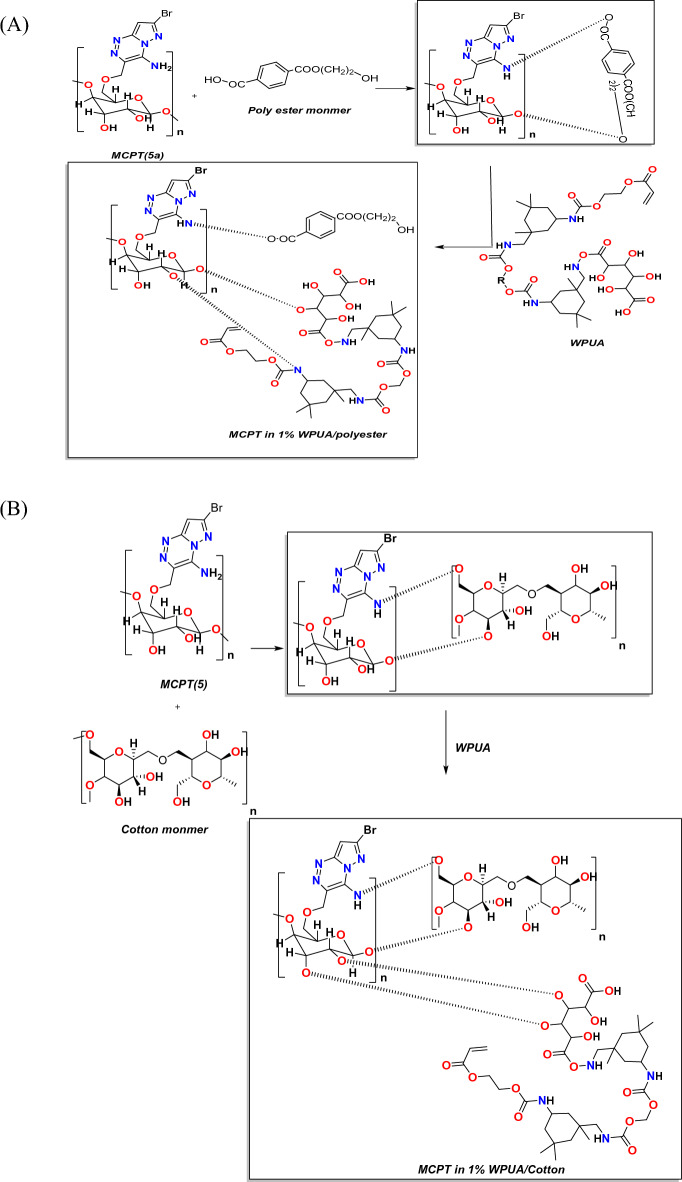

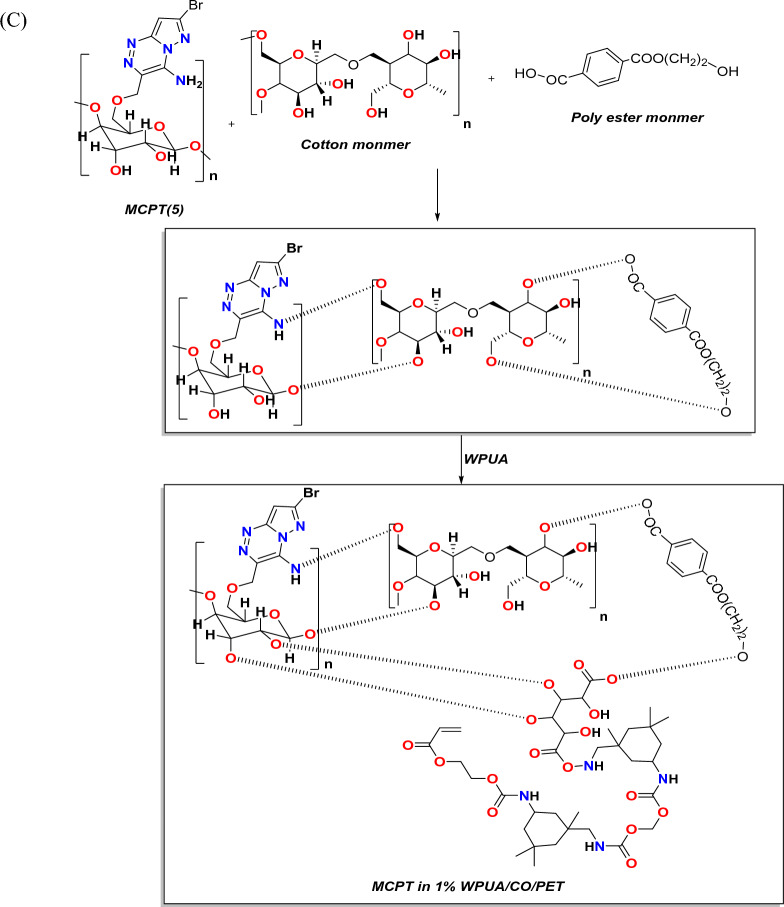
(**A**), (**B**), and (**C**) Proposed mechanism of interaction of MCPT in 1%WPUA with different fabrics.

Cotton fabric consisted of cellulose united with good dye adhesion ability owing to the presence of –OH functionality^50^. The interaction between MCPT and cellulosic fabric may be achieved through the formation of intermolecular hydrogen bonding and these results were showed in the following proposed Fig. 7A,B,C) which showed the presence of amino group and OH of cellulose in MCPT dye can be easily interact with the fabrics in the OH of polyester, Cotton and CO/PET fabrics which gave stability and make staining of the dye on the fabrics.

**Figure 8:**
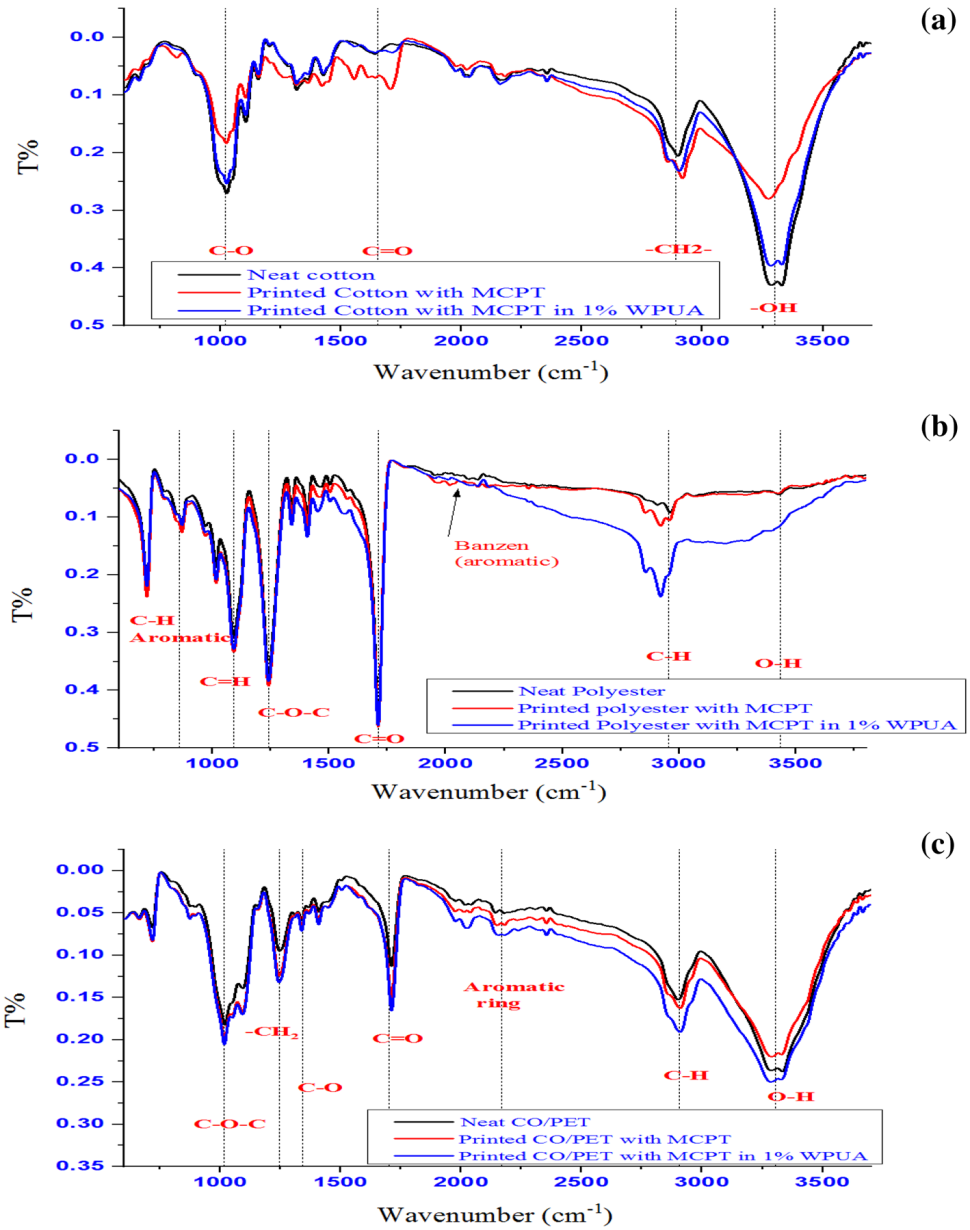
IR spectrum of printed fabric.

The FT-IR spectral of printed fabrics are illustrated in Fig. 8. The changes that occurred in the MCPT printed cotton spectrum may be a result of the interaction between cellulose–OH and MPCT–NH_2_, though hydrogen bond formation led to a reduction of absorption intensity bands around 3330 and 3270 cm^−1^ in the neat cotton fabric spectrum. The unbounded OH in WPUA could be the reason for the additional increase in the-OH absorption band in the FT-IR spectrum of MCPT in WPUA printed cotton fabric. Alternatively, the polyester fabric has no reactive functional group to interact with colorant material. The water-insoluble nonionic MCPT may be worked as a dispersed dye and MCPT particles can interact with the polyester chains due to their low dissociation in water. At the temperature of 130 °C or higher, thermal agitation reasons the polyester's molecular structure to developed more amorphous, allowing MCPT particles to enter and adhere to the polyester fiber by van der Waals and dipole forces^51^.

**Figure 9:**
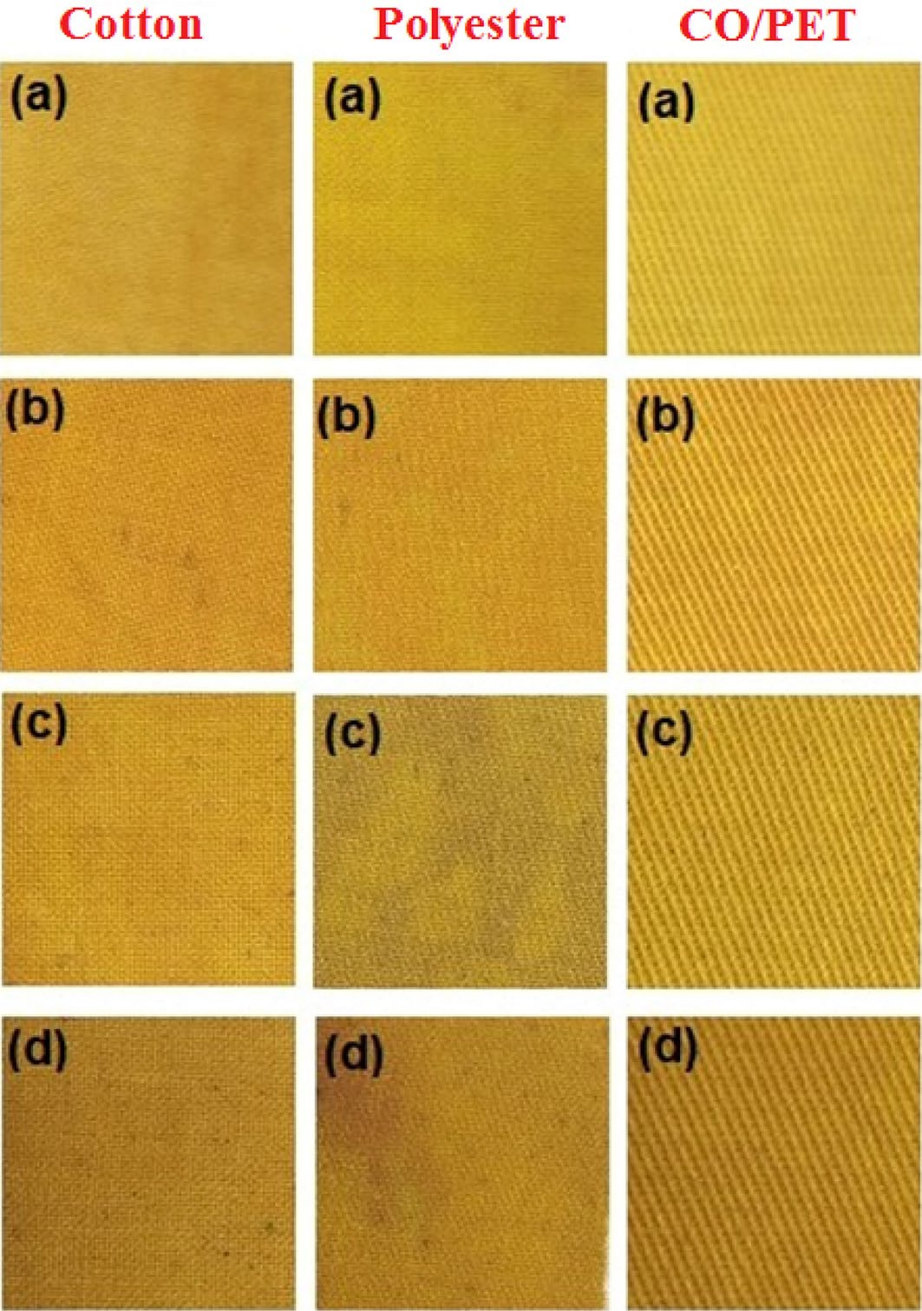
Photographs of printed cotton, polyester, and Co/PET fabrics with MCPT (**a**), MCPT (2 min ultra-sonication) (**b**), 1% WPUA (**c**), and 1% WPUA (2 min ultra-sonication) (**d**), respectively.

IR spectrum of neat and printed polyester fabrics may help to clarify this interaction. The fig demonstrated that there was virtually no difference in the spectrum bands of MCPT printed polyester from a neat one because the concentration of absorbed MCPT in the polyester fabric was too low to exhibit its excitation in the spectrum^52^. However, the spectra of printed polyester by WPUA dispersion of MCTP revealed a broad absorption band. This might be related to WPUA's unbounded OH group. Furthermore, the situation is interesting somewhere in CO/PET fabric, because consists of both callouses and polyester fiber, where the two proposed mechanisms of interaction are predicted to occur, as seen in the spectra of neat CO/PET, MCTP, and MCTP in WPUA printed fabrics, as presented in Fig. 8. The Fig. 9 exhibited the photographic photos of printed cotton, polyester and specially blended (cotton polyester blend 50:50) fabrics by MCPT and MCPT dispersed in 1% WPUA (before and after ultra-sonication) using silk screen technique. The color strength (K/S) and CIE lab color parameters of printed fabrics were evaluated and illustrated in Table 2. According to the data, all printed fabrics with **MCPT** have a significant K/S value. Some improvement in K/S was observed with all printed fabrics after printing with ultrasonic dispersion **MCPT** compared to those printed MCPT, which could be accredited to the reduction in **MCPT** particle size due to the ultrasonic effect leading to more color penetration. On the other hand, the printed fabrics with **MCPT** dispersed in 1% WPUA introduced more enhancement in K/S of prints due to the binding properties of WPUA (forming a thin film covering the colorant particles after curing and restricting their release or discharge), while there was no notable improvement in the value of the blend sample's color depth^53^. Although applying an ultrasonic dispersion of (MCPT in 1% WPUA) to the printing paste did not enhance the K/S values of polyester fabric, it achieved improve the K/S values of cotton and blend samples. All these changes in color and hue are expressed by changes in the values of L*, a*, and b*. The analysis of color parameters a*, and b* of printed MCPT samples revealed that all printed samples exhibit a yellowish-green shift, which is compatible with the samples' apparent color. Furthermore, the L values revealed that all samples have values greater than 50, indicating that they are in the lighter area, in differing degrees depending on the intensity of the color on the printed sample's surface.

**Table 2:** Color strength (K/S) and CIE lab color parameters of printed fabrics.

Printed Sample		K/S λ_max_ = 370 nm	L*	a*	b*
MCPT	Cotton	12.60	58.81	17.68	50.77
	Polyester	9.90	68.33	16.10	51.19
	CO/PET	10.17	66.71	16.40	52.22
MCPT (2 min sonication)	Cotton	13.13	61.17	12.19	54.68
	Polyester	10.81	65.83	13.11	50.24
	CO/PET	11.37	64.86	12.16	53.21
MCPT in 1% WPUA	Cotton	14.37	57.12	14.47	50.05
	Polyester	12.05	62.04	14.29	51.95
	CO/PET	10.73	67.68	12.57	56.23
MCPT in 1% WPUA (2 min ultra-sonication)	Cotton	15.56	54.91	9.25	43.25
	Polyester	9.62	65.25	10.47	46.06
	CO/PET	12.17	64.23	10.30	50.94

### Ultraviolet protection factor (UPF) evaluation

Table 3 demonstrates the ultra-violet shielding activity of printed fabrics by MCPT and MCPT in 1% WPUA before and after ultra-sonic action as UPF values. The synthesized MCPT has excellent UV-blocking activity with all printed fabrics, as shown by the UPF values, according to the findings. However, it appeared that the application of ultrasonic resulted in a reduction of UPF values of both polyester and CO/PET prints. The UV capping activity of the acrylate portion of polyurethane acrylate may be responsible for the enhancement of UPF values of the printed fabrics by MCPT in 1% WPUA^46^. Ultrasonic action of MCPT in 1% WPUA was also shown to reduce the UV protection value of cotton and polyester prints.

**Table 3:** UPF evaluation values of printed fabrics by MCPT.

Printed sample		UPF
MCPT	Cotton	66.0
	Polyester	81.3
	CO/PET	276.0
MCPT (2 min sonication)	Cotton	74.7
	Polyester	47.2
	CO/PE	195.2
MCPT in 1% WPUA	Cotton	395.1
	Polyester	126.7
	CO/PET	276.3
MCPT in 1% WPUA (2 min sonication)	Cotton	278.6
	Polyester	62.3
	CO/PET	289.3

### Fatness properties of dye

The fastness properties of printed fabrics are represented in Table 4. The results were printed samples with MCPT (2 min sonication) with good light fastness. Better light fastness was observed with samples printed by MCPT in 1% WPUA (the samples become darker). The washing and perspiration fastness showed values ranged from good to very good with printed fabrics with MCPT. Further improvement in fastness properties was observed with the fabrics printed by MCPT dispersed in 1% WPUA. This may be clarified by the fact that the printing process is a surface application and its major drawback is related to absorption like in washing and rubbing, and WPUA works as a film former macromolecule that taps the colorant particles, So the formed film resisted the surface abrasion and limited the colorant release, which resulted in an improvement in fastness properties and coloration performance^53^.

**Table 4:** Fatness properties of printed fabrics by MCPT.

Samples		Light	Rubbing		Washing			Perspiration					
								Acidic			Alkaline		
					St.		Alt.	St.		Alt.	St.		Alt.
			dry	wet	cotton	wool		wool	cotton		cotton	wool	
MCPT (2 min sonication)	Cotton	4–5	3	3	3–4	3	3–4	3	2–3	4	3	3	3–4
	Polyester	5	2	2	3	3	4	3	2–3	4	2–3	2	3
	CO/PET	5–6	3	3	3	3	3–4	3	2–3	4	3	2–3	3–4
MCPT—in 1% WPUA	Cotton	6	4	3–4	3–4	3–4	3–4	3	2–3	4	3	2–3	4
	Polyester	6	2–3	2	3–4	3–4	4–5	4	3	4	3	2–3	3
	CO/PET	6	3	3–4	3–4	3–4	4–5	3–4	3	4–5	3–4	3–4	4–5

Where, Alt = alteration St. = staining.

## Biological action

### Antibacterial investigation

**Figure 10:**
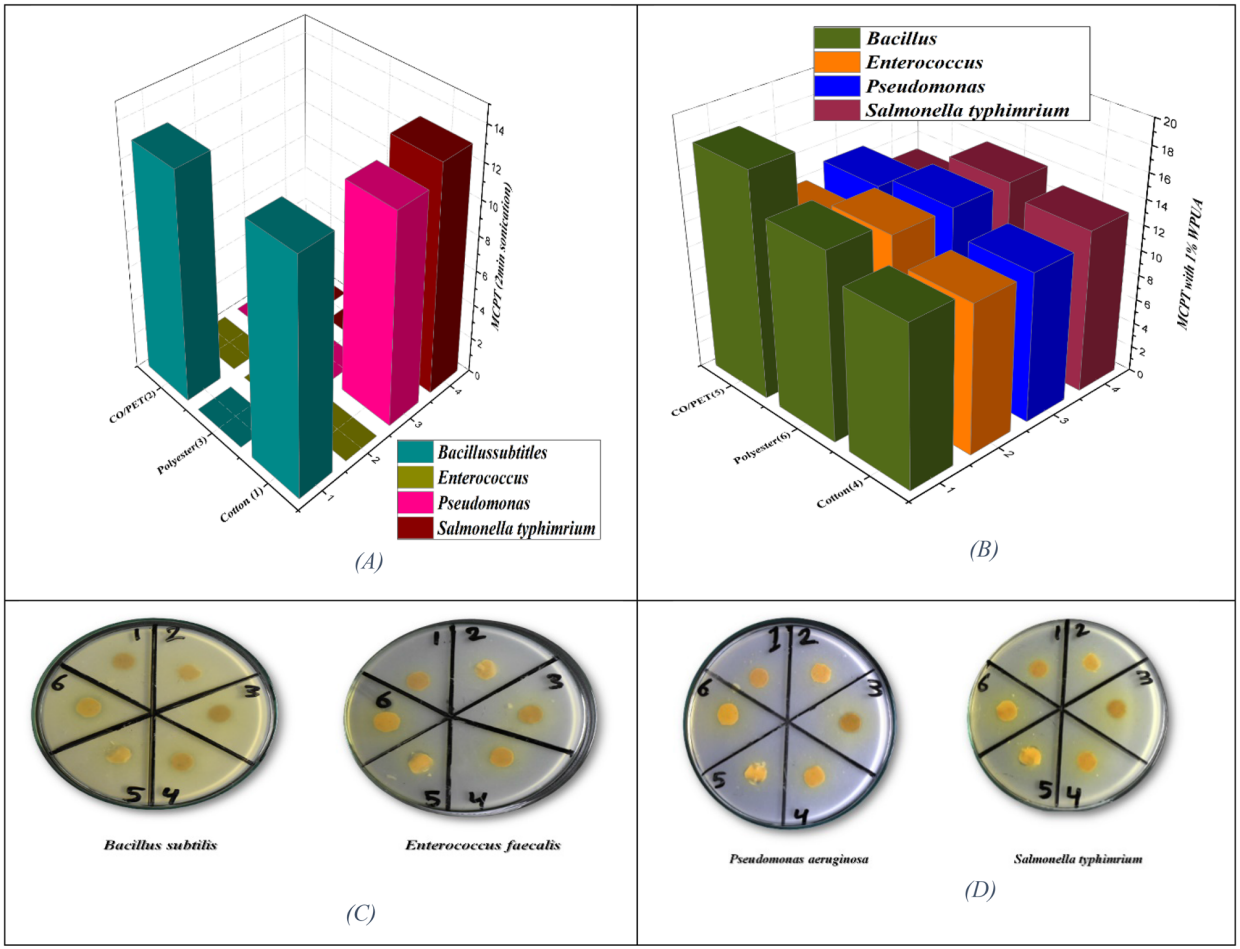
Antibacterial diagram and dishes of biological activities.

The antibacterial action of fabrics was established compared to inhibitory properties on the development of *G*+ and *G−* bacterial strains as displayed in Table 5. The presence of NH_2_ on MCPT and the OH groups in cotton increases the activity of antibacterial activity, so the action with cotton exhibited higher activity compared to all strains, while it exhibited less activity with polyester and a cotton/polyester blend, and no activity with polyester. Furthermore, the presence of MCPT with 1% dispersed WPUA polymer revealed that it increased the activity of printed fabric and showed excellent activity with all types of antibacterial strains. Also, the presence of polyurethane with MCPT increases the efficacy of polyurethane against antimicrobials, which depends on the nature and hydrophilicity of WPUA, which provides a good opportunity for friendly interaction with aqueous germ suspension, that improves the presentation of polyurethanes^54^. The result of the analysis revealed that the printed fabric efficiency against microbial species (gram-negative and positive bacteria) varies depending on the bacterial strain. However, the highest anti-microbial effect was detected against Bacillus subtilis, *as* shown in Table 5 and Fig. 10

**Table 5:** The antibacterial activity screening of the printed fabrics.

Sample of Fabric		Inhibition zone diameter (mm/cm Sample)			
		Bacterial species			
		G^+^		G^−^	
		Bacillus subtilis	Enterococcus faecalis	Pseudomonas aeruginosa	Salmonella typhimrium
MCPT (2 min sonication)	Cotton (1)	13	0.0	12	13
	Polyester(3)	0.0	0.0	0.0	0.0
	CO/PET(2)	13	0.0	0.0	0.0
MCPT in 1% WPUA	Cotton(4)	13	13	12	12
	Polyester(6)	15	14	14	14
	CO/PET (5)	18	11	13	12

### Docking investigation

**Figure 11:**
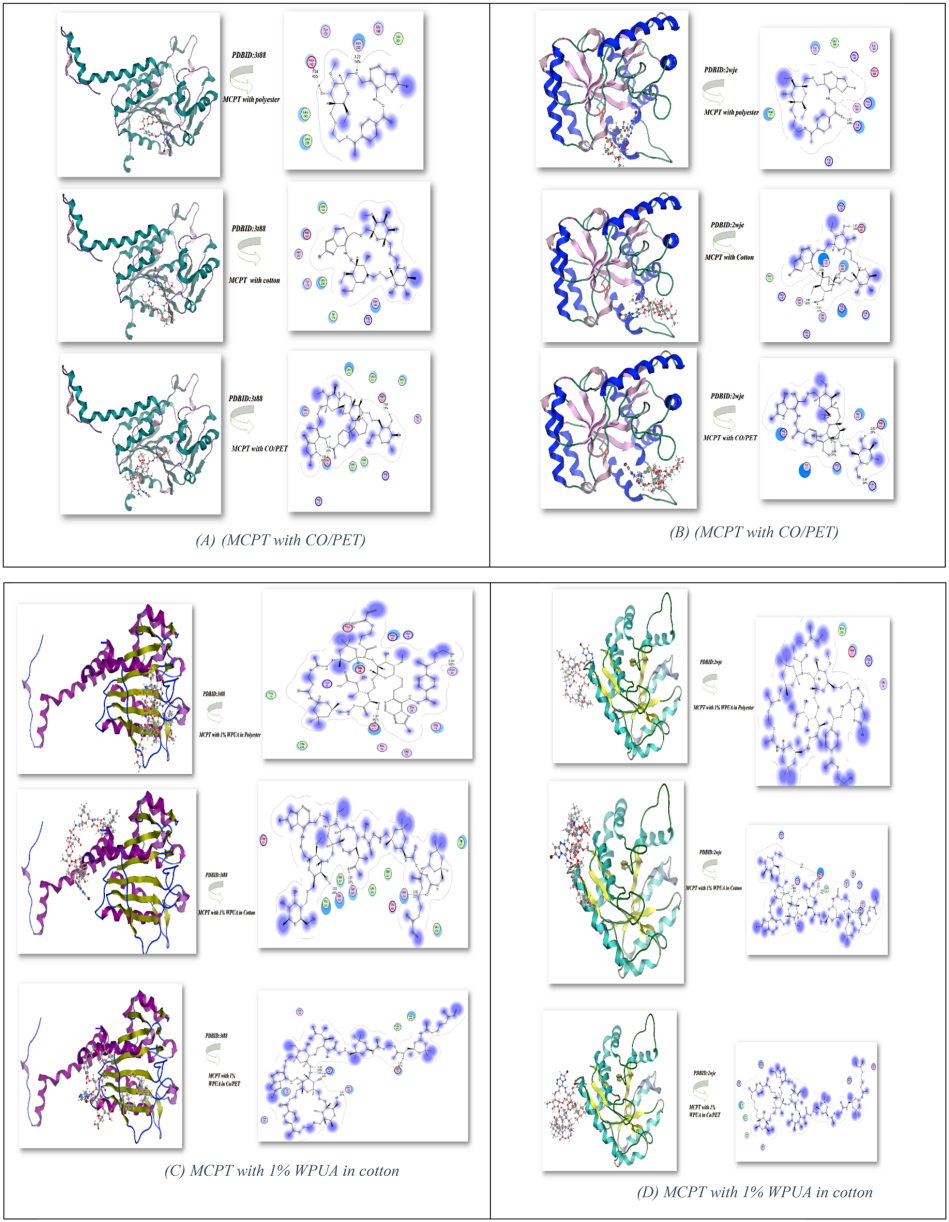
Docking stimulation of dispersant MCPT and 1%WPUA with different fabric.

Docking simulation of MCPT, 1%WPUA, and staining compounds with different fabrics was utilized using the MOE program^38^ to recognize the antibacterial action to be well-matched with experimental investigation. These monomers of compounds were docked with Crystal structure of Escherichia coli MenB in complex with substrate analogue, OSB-NCoA (**PDBID**: 3t88)^39^ and the Crystal structure of the tyrosine phosphatase Cps4B from Streptococcus pneumonia TIGR4 (**PDBID**: 2wje)^40^ as demonstrated in Table 6 and Fig. 11 It was observed that the moderate required energy between MCPT/polyester fabric monomer with **PDBID: 3t88** showed -12.422 kcal/mol and shortage bond length 1.58–3.22 Å and higher binding with cotton with the binding energy − 13.7446 kcal/mol due to electrostatic hydrogen bonding interaction and shortage length 2.05 Å and the lowest energy with CO/PET with − 11.156 kcal/mol and length 1.56 Å as showed in Fig. 11A also, binding energy of these dispersant fabric MCPT/polyester with **PDBID:2wje** showed − 11.8775 kcal/mol and length 2.62 Å and MCPT/Cotton showed excellent binding with − 13.0344 kcal/mol with length 1.47Å while the least binding MCPT with CO/PE − 10.8244 kcal/mol and shortage length 2.45 Å as displayed in Fig. 10B and these result compatible with experimental study. Furthermore, the docking investigation of dispersant MCPT with 1%WPUA with different fabrics which showed the most binding energy of **PDBID:**3t88 and **PDBID:**2wje with MCPT with 1% WPUA and CO/PET (− 14.325 kcal/mol, − 12.632 kcal/mol; respectively) and length range 1.95–3.16 Å and showed different amino acid linkage, also the binding energy of MCPT/1%WPUA with cotton showed − 13.945, − 12.096 kcal/mol and length 1.67–3.07 Å showed different hydrogen bonding with –OH of cotton fabric; respectively, while the fabric of polyester showed the less binding interaction as displayed in Fig. 11C,D and Table 6.

**Table 6:** Docking simulation of fabrics to **PDBID:3t88** and **PDBID: 2wje**.

Escherichia coli (PDB:3t88)				Streptococcus pneumoniae (PDB:2wje)			
	Energy affinity (kcal/mol)	Distance(Å)	Amino acids		Energy affinity (kcal/mol)	Distance(Å)	Amino acids
MCPT with fabric							
MCPT with polyester	− 12.422	1.58, 3.22 Å	Asp 142, Asn 202, Val 201, Gly 199, Tyr 170	MCPT with Polyester	− 11.8775	2.62 Å	Arg 206, Asn 162, Arg 139, Asp 199, His 166, Met 180, Tyr 177, Ser 165
MCPT with cotton	− 13.7446	2.05 Å	Asp 142, Asn 237, Leu 143, Leu 236, Tyr 170	MCPT with Cotton	− 13.0344	1.47, 1.49, 2.94, 2.61 Å	Tyr 177, Gly 205, Ser 165, Arg 206, Lys 171, Asp 204, Arg 176
MCPT with CO/PET	− 11.1568	1.69,1.56 Å	Asn 224, Leu 236, Leu 229, Met 221, Asp 142, Leu 232, Leu 143, Leu 236	MCPT with CO/PET	− 10.8244	2.69, 2.82, 2.45 Å	Asp 204, Lys 171, Arg 176, Gly 206, Tyr 177, Arg 206
MCPT with1% WPUA and fabric							
MCPT with 1% WPUA and polyester	− 12.632	1.87, 2.82 Å	Asn 40, Thr 38, Gly 78, Glu 211, Gln 26	MCPT with 1% WPUA and polyester	− 11.521	2.06 Å	Gly0, Arg 35, Asp 72, Met 239
MCPT with1% WPUA and cotton	− 13.945	1.5, 2.63 Å	Gln 223, Met 227, Ser 225, Pro 226, Leu 222, Asn 224, Met 221	MCPT with1% WPUA and cotton	− 12.096	1.67, 3.21, 1.38, 3.07 Å	Lys 23, Glu 68, Val 69, Tyr 30, Gly 33, Arg19
MCPT with 1% WPUA and CO/PET	− 14.325	2.69, 3.16, 2.9 Å	Asn 149, Arg 45, Tyr 125, Gln 165	MCPT with 1% WPUA and CO/PET	− 12.632	1.95 Å	Asn 100, Asp 72, Val 238, Glu 64, Met239, Arg 236

## Computational investigation

### Physical characterization

**Figure 12:**
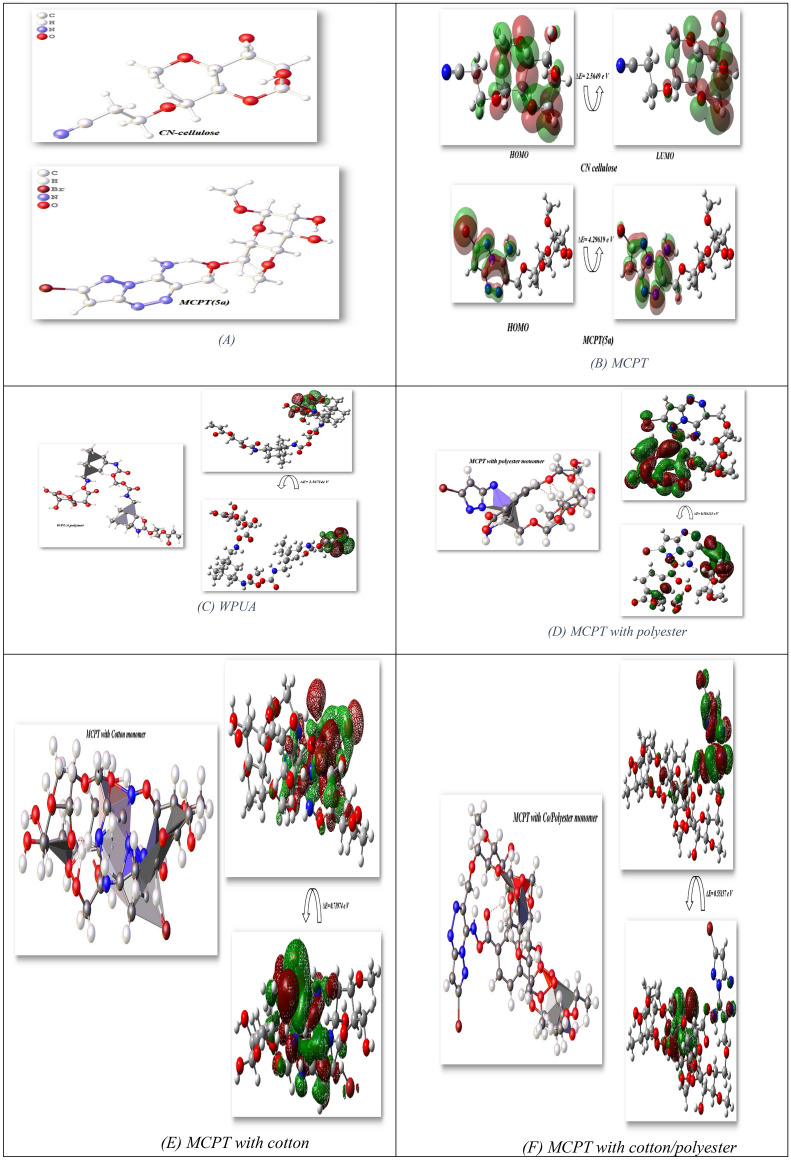
Optimized, HOMO–LUMO of MCEC, MCPT, and MCPT with different fabric.

In this investigation, optimization of Br-cellulosic dye with different fabric monomers utilized Gaussian(09)^41^ finished DFT/B3LYP/6-31(G) level. Additionally, the physical features of **MCPT(5a), MCPT/polyester**, **MCPT/Cotton**, and **MCPT/Co/PET** monomers were relating to (σ) softness^55^, (χ) electronegativity’s^56^, (ΔN_max_) electronic charge^57^, (η) hardness, (ω)^58^ electrophilicity^59^, (S) softness^60^, and chemical potential^61^, from the equations [1–8] which were listed in Table 7 and Fig. 12^62^.

**Table 7:** Physical characterization parameters of different cellulosic fabric structures.

DFT/B3LYP/6-31G (d)						
Physical parameters	MCEC(3)	MCPT(5a)	MPUA	MCPT with Polyester monomer	MCPT with cotton monomer	MCPT with CO/polyester monomer
ET (au)	− 896.44 (au)	− 3844.317 (au)	− 2789.6685 (au)	− 4599.429 8(au)	− 5171.7643 2(au)	− 5935.706 2(au)
E_HOMO_ (eV)	− 4.9116938	− 5.98573	− 4.554949	− 3.259677	− 1.2302364	− 2.634899
E_LUMO_ (eV)	− 7.476659	− 1.68956	− 1.9878	− 3.843910	− 1.94998	− 3.186478
ΔE (ev)	2.5649	4.29619	2.5671	0.584233	0.71974	0.551579
µ (Debye)	1.8320	5.8438	11.6670	13.3651	13.9600	18.4068
χ (eV)	6.194	3.838	3.271	3.552	1.590	0.2911
η (eV)	− 1.282	2.148	1.284	0.292	0.360	0.276
σ (eV)	− 0.780	0.466	0.779	3.423	2.779	3.626
Pi (eV)	− 6.194	− 3.838	− 3.271	− 3.552	− 1.590	− 2.911
S (eV)	− 0.390	0.233	0.390	1.712	1.389	− 1.813
ω (eV)	− 14.958	3.428	4.169	21.593	3.513	− 15.360
ΔN max	4.831513	1.78677	2.54750	3.42465	4.41666	10.54710

**Table:** 

$\Delta E = E_{LUMO} - E_{HOMO}$	[1]	$\chi = \frac{{ - \;(E_{HOMO} + E_{LUMO} )}}{2}$	[2]
$\eta = \frac{{(E_{LUMO} - E_{HOMO} )}}{2}$	[3]	σ = 1/η	[4]
${\text{Pi}} = -$Ӽ	[5]	S = 1/2 η	[6]
ω = Pi^2^/2	[7]	ΔN max = − Pi/η	[8]

Besides, the enhanced structures exhibited non-planarity used DFT/B3LYP/6-31 (G) level which showed **MCEC(3)** less energy with (− 896.44 au), although the reactivity increase to − 3844.317 au attributable to occurrence of Bromine in a para location which extent the stability of compound **MCPT(5a),** as well as the µ of **MCEC (3)** offered the least 1.8320D with can simply energy separation and gave it ability to react again and increased in **MCPT (5a)** 5.8438D which gave it stability. Electronegativity (χ) defined the attraction of atom with pair of electrons and observed **MCEC (3)** exhibited a high charge with 6.194 eV as a result of the occurrence of cyanide group and has ability to react again. Correspondingly, hardness η(eV) designates the transformation of electron cloud density in the structure and presented the small range of **MCEC (3)** with − 1.282 eV to modification of electron cloud because of cyanide. chemical potential of heterocyclic attached to fabric and the facility to absorb more energy in range − 2.498 and − 6.194 eV which presented capability to accumulation energy inside them. ω directed electrophilic character and electron movement among donor and acceptor so, the **MCPT(5a)** displayed that higher electophiphilc atmosphere to captivate electrons with 3.428 eV, while **MCEC (3)** showed less ω with − 14.958 eV to not absorb more electrons and established their reactivity to formation the new heterocycles. The great gap will have high stability and low reactivity, although a little gap will have low stability and high reactivity^63,64^, as demonstrated in Fig. 11A. HOMO–LUMO calculated at the B3LYP/6-31G(d, p) basis set for glycoside compound **MCEC (3)** exhibited band energy gap = 2.5649(eV), though distribution of electrons in **MCPT(5a)** attached to *p*-Br benzene as a result of withdrawing character and higher stability of this dye with band energy gap 4.29619 (eV) as exhibited in Fig. 12B. The optimization of WPUA showed energy − 2789.6685 (au) and the difference in HOMO–LUMO was 2.5671 eV (59.19872 kcal/mol) and it showed the stability of this polymer and its dipole moment 11.6670D and indicate the easily charge separation as displayed in Fig. 12C and its electronegativity’s and chemical hardness were 3.271 (eV) (75.431 kcal/mol), 1.284 (eV) (28.7796 kcal/mol); respectively and can easily to interact and make hydrogen bonding with MCPT, which confirmed experimental elucidation of this dispersant with each others^65,66^.

Moreover, the interaction of MCPT of polyester, cotton, and Co/PET fabrics was investigated in Table 7 and Fig. 12D,E,F respectively. The reactivity of Co/PET showed more binding energy with − 5935.7062 (au), and MCPT/Cotton with −5171.76432 (au) and less interaction of MCPT/polyester (− 4599.4298 au) and all of the interaction of MCPT with all fabric showed band gap energy between FMO is > 1 and take range 0.55–0.71974 (eV) and showed the reactivity of this fabric and can easily to stained in the fabric. The dipole moment of Co/PET showed the highest value of 18.4068D can easily of the separation of charge. Furthermore, (χ) electronegativity’s MCPT/polyester showed a high value with 3.552 eV can indicate interaction again and the MCPT/Co/PET showed the lowest value with 0.2911 eV due to more interaction.

Also, hardness η(eV) specifies the amount of resistance electron cloud density change in the structure and presented the low range of all fabrics, and take a range of 0.276–0.360 eV and increasing value in softness for all fabrics with range 2.779 eV for cotton and 3.423–3.626 eV for polyester and Co/PET; respectively. The chemical potential (Pi) of this dispersant fabrics showed the least value with MCPT/cotton and indicated to staining of cotton fibers surface, and MCPT/polyester and MCPT/Co/PET with range − 3.552 eV, − 2.911 eV; respectively. The HOMO–LUMO electron cloud mostly appeared on the interaction between them and showed hydrogen bonding interaction^67^.

**Figure 13:**
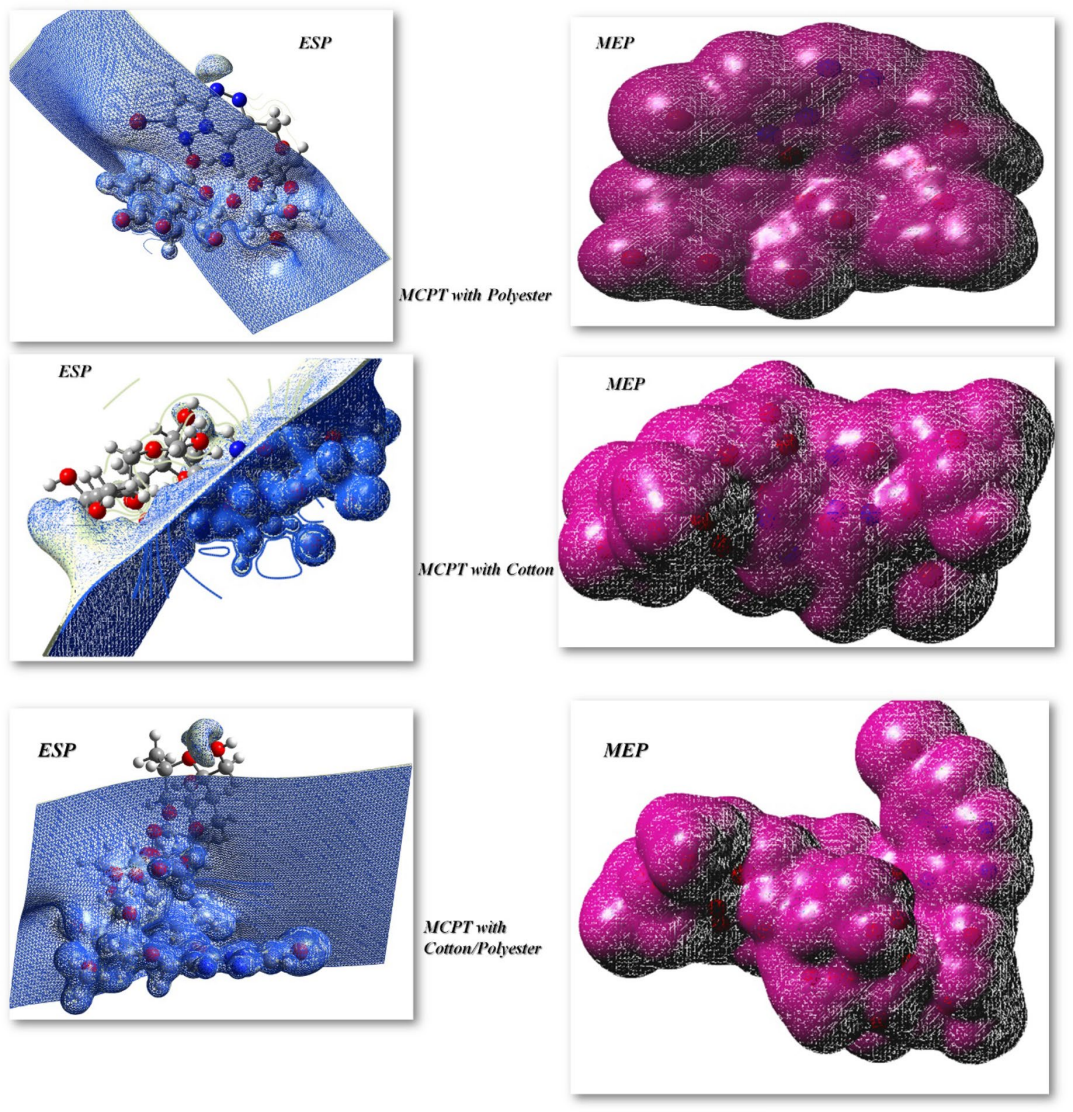
MEP and ESP of MCPT with different fabric.

Additionally, the hydrogen bonding interaction which introduced through electrophilic and nucleophilic active sites and were determined utilized ESP, MEP, which investigating the molecular performance and showed polarity of molecule with active sites in charge distribution in 3D and their know physicochemical parameters^68,69^. To expect active sites for electrophilic and nucleophile positions in the compound, molecular electrostatic potentials (MEP) were calculated via B3LYP/6-31G(d,p) basis set, as displayed in Fig. 13. The common of the −ve regions and +ve charges of most of the interaction bond between cellulosic compound MCPT with polyester, cotton, and Co/PET were dispersed exclusive the pocket which designates their and established the biological estimation of them and dispersion of MCPT on the surface of the fabric as displayed in Fig. 13.

Additionally, the hydrogen bonding interaction which introduced through electrophilic and nucleophilic active sites and were determined utilized ESP, MEP, which investigating the molecular performance and showed polarity of molecule with active sites in charge distribution in 3D and their know physicochemical parameters^68–70^. To expect active sites for electrophilic and nucleophile positions in the compound, molecular electrostatic potentials (MEP) were calculated via B3LYP/6-31G(d,p) basis set, as displayed in Fig. 13. The common of the −ve regions and +ve charges of most of the interaction bond between cellulosic compound MCPT with polyester, cotton, and Co/PET were dispersed exclusive the pocket which designates their and established the biological estimation of them and dispersion of MCPT on the surface of the fabric as displayed in Fig. 13.

## Conclusion

In this elucidation, we synthesized the novel Br-cellulosic dye fused heterocycles through the reaction of C≡N cellulose with diazonium salts to give MCPT_dye_ which was confirmed through different spectral analysis, also MCPT dispersion in 1% WPUA improved its dispersion stability. the prepared MCPT_dye_ effectively colored all of the polyester, cotton, and CO/PET fabrics with considerable color depth and good fastness properties. In the printing past, the application of MCPT_dye_ in 1% WPUA dispersion improved the color fastness properties of all printed fabrics. All MCPT _dye_-printed fabrics demonstrated excellent UV-blocking activity. Furthermore, MCPT_dye_ dispersion in 1% WPUA improved UPF rating of printed fabrics. Antimicrobial activity was examined in MCPT_dye_ in 1% WPUA printed fabrics and showed excellent activity. Furthermore, docking stimulation and optimization of these fabrics with MCPT revealed a high level of interaction, confirming the experimental result.

## Data Availability

All data generated or analyzed during this study are included in this published article.
